# Detection of *ATM* germline variants by the p53 mitotic centrosomal localization test in *BRCA1/2*-negative patients with early-onset breast cancer

**DOI:** 10.1186/s13046-016-0410-3

**Published:** 2016-09-06

**Authors:** Andrea Prodosmo, Amelia Buffone, Manlio Mattioni, Agnese Barnabei, Agnese Persichetti, Aurora De Leo, Marialuisa Appetecchia, Arianna Nicolussi, Anna Coppa, Salvatore Sciacchitano, Carolina Giordano, Paola Pinnarò, Giuseppe Sanguineti, Lidia Strigari, Gabriele Alessandrini, Francesco Facciolo, Maurizio Cosimelli, Gian Luca Grazi, Giacomo Corrado, Enrico Vizza, Giuseppe Giannini, Silvia Soddu

**Affiliations:** 1 Unit of Cellular Networks and Molecular Therapeutic Targets, Department of Research, Advanced Diagnostic, and Technological Innovation, Regina Elena National Cancer Institute – IRCCS, Via Elio Chianesi 53, 00144 Rome, Italy; 2 Istituto Pasteur-Fondazione Cenci Bolognetti, Department of Molecular Medicine, University La Sapienza, Rome, Italy; 3 Endocrinology Unit, Department of Clinical and Experimental Oncology, Regina Elena National Cancer Institute – IRCCS, Rome, Italy; 4 Department of Molecular Medicine, University La Sapienza, Rome, Italy; 5 Department of Clinical and Molecular Medicine, University La Sapienza, Laboratorio di Ricerca Biomedica, Fondazione Università Niccolò Cusano per la Ricerca Medico Scientifica, Rome, Italy; 6 Radiotherapy Unit, Department of Research, Advanced Diagnostic, and Technological Innovation, Regina Elena National Cancer Institute – IRCCS, Rome, Italy; 7 Medical Physics Unit, Department of Research, Advanced Diagnostic, and Technological Innovation, Regina Elena National Cancer Institute – IRCCS, Rome, Italy; 8 Toracic Surgery Unit, Department of Clinical and Experimental Oncology, Regina Elena National Cancer Institute – IRCCS, Rome, Italy; 9 Hepato-pancreato-biliary Surgery Unit, Department of Clinical and Experimental Oncology, Regina Elena National Cancer Institute – IRCCS, Rome, Italy; 10 Gynecological Oncology Unit, Department of Clinical and Experimental Oncology, Regina Elena National Cancer Institute – IRCCS, Rome, Italy; 11 Department of Experimental Medicine, Sapienza University of Rome, Policlinico Umberto I, Viale Regina Elena, 32400161 Rome, Italy

**Keywords:** *ATM* cancer susceptibility gene, Early-onset breast cancer, BRCA1/2, p53-mitotic centrosomal localization (p53-MCL)

## Abstract

**Background:**

Variant *ATM* heterozygotes have an increased risk of developing cancer, cardiovascular diseases, and diabetes. Costs and time of sequencing and *ATM* variant complexity make large-scale, general population screenings not cost-effective yet. Recently, we developed a straightforward, rapid, and inexpensive test based on p53 mitotic centrosomal localization (p53-MCL) in peripheral blood mononuclear cells (PBMCs) that diagnoses mutant *ATM* zygosity and recognizes tumor-associated *ATM* polymorphisms.

**Methods:**

Fresh PBMCs from 496 cancer patients were analyzed by p53-MCL: 90 cases with familial *BRCA1/2-*positive and -negative breast and/or ovarian cancer, 337 with sporadic cancers (ovarian, lung, colon, and post-menopausal breast cancers), and 69 with breast/thyroid cancer. Variants were confirmed by *ATM* sequencing.

**Results:**

A total of seven individuals with ATM variants were identified, 5/65 (7.7 %) in breast cancer cases of familial breast and/or ovarian cancer and 2/69 (2.9 %) in breast/thyroid cancer. No variant *ATM* carriers were found among the other cancer cases. Excluding a single case in which both *BRCA1* and *ATM* were mutated, no p53-MCL alterations were observed in *BRCA1/2*-positive cases.

**Conclusions:**

These data validate p53-MCL as reliable and specific test for germline *ATM* variants, confirm *ATM* as breast cancer susceptibility gene, and highlight a possible association with breast/thyroid cancers.

**Electronic supplementary material:**

The online version of this article (doi:10.1186/s13046-016-0410-3) contains supplementary material, which is available to authorized users.

## Background

Biallelic mutations in the *ATM* gene cause Ataxia-telangiectasia (A-T), a rare autosomal recessive multisystemic disorder characterized by progressive cerebellar ataxia, immune defects, insulin-resistant diabetes, radiosensitivity, and high risk for malignancy [1, 2]. The *ATM* gene spans approximately 160 Kb of genomic DNA containing 66 exons [3] and encodes ATM protein, a serine/threonine kinase mainly involved in DNA damage response pathways following DNA double strand breaks [4]. An enormous number of mutations (more than 600) can occur in the coding and noncoding regions of the *ATM* gene without hotspots [5]. In A-T patients, the large majority of ATM mutations are protein-truncations or splice-junction variants that can be easily distinguished by the numerous ATM polymorphisms [6, 7].

Heterozygous carriers of variants in the *ATM* gene (from here on, ATM carriers) are usually asymptomatic and largely considered healthy carriers. However, they have been reported to be more sensitive to ionizing radiation and susceptible to ischemic heart disease, diabetes, and cancer, particularly of the breast, but also digestive tract and lung [2, 8]. Many association studies have been performed on breast cancer susceptibility. Initially, epidemiological studies on relatives of A-T patients revealed a two to fivefold increased in the risk of breast cancer for female obligate ATM carriers [9]. The increased risk of breast cancer in ATM carriers was then confirmed by direct *ATM* sequencing in breast cancer cases compared to controls [10] and *ATM* is now considered a moderate-penetrance cancer susceptibility gene in *BRCA1/2*-negative patients with familial early-onset breast cancer [11]. Along with A-T associated mutations, several ATM screenings in cancer patients identified missense ATM variants, particularly amino acid substitutions that are not expected to be associated with A-T [12]. However, discrimination of these ATM variants from ATM polymorphisms and their contribution to health risks is still controversial. In addition, distinguishing between deleterious and neutral ATM alterations is required to allow the definition of standard-of-care clinical guidelines for the management of ATM carriers and their families [11]. Systematic review and meta-analysis of *ATM* sequencing data have been conducted to evaluate the health risks for parents and siblings of A-T patients, but similar large-scale screenings for ATM carriers in the general population by direct sequencing are not cost-effective yet [13].

Recently, we have developed a rapid, reliable and non-expensive test based on the ATM-dependent p53-mitotic centrosomal localization (p53-MCL) that clearly discriminates ATM carriers of A-T mutations and at least some of the ATM cancer predisposition variants in lymphoblastoid cell lines (LCLs) and PBMCs. At variance with other diagnostic tests, the p53-MCL assay does not measure a continuous quantitative variation (*e.g*., radiosensitivity, ATM protein levels, phosphorylation of ATM targets) but a “binary” outcome. Indeed, at the single cell level, p53 does or does not localize at the centrosomes while, at the cell-population level, the number of cells showing one or the other phenotype allows to unambiguously diagnose A-T homozygotes and ATM carriers [14].

In a preliminary set up and validation of the p53-MCL test, we showed that it is highly sensitive, specific, and precise. In particular, we assessed the specificity by analyzing LCLs from monogenic disease carrying mutations in a series of DNA-damage related factors, such as MRE11, NBS1, SMC1A, WRN, ATR, FANC-A, and p53 [14]. In addition, p53-MCL test revealed 7 ATM carriers among 80 sporadic breast cancer patients. By direct ATM sequencing of 3 of these carriers, we identified the cancer-prone intronic c.8786 + 8A > C variant [15] in one patient and the c.2572 T > C (p.F858L) missense mutation [16] in other two patients. No ATM carriers were observed in a comparable cohort of healthy donors [14]. These data support p53-MCL as promising candidate test for cost-effective, large-scale screenings of ATM carriers.

Here, we examined validity and specificity of p53-MCL analyzing 15 LCLs from familial breast and ovarian cancer cases and fresh PBMCs from a total of 496 cases including *BRCA1/2*-positive and -negative familial breast and ovarian cancer and different sporadic cancers.

## Methods

### Patients

A total of 496 cancer patients were enrolled from 2010 to 2015 at three different Italian institutes: Policlinico Umberto I (University La Sapienza), Sant’Andrea Hospital (University La Sapienza), and Regina Elena National Cancer Institute - IRCCS. In particular, according to previously described criteria [17], we selected 90 unrelated families affected with breast and/or ovarian cancer after interview at the Hereditary Tumors Counseling Centre of the Policlinico Umberto I. Pre-test counseling was performed by an expert cancer risk counselor and the probands analyzed in this study belonged to different high-risk classes. During the genetic counseling, we calculated the a priori probability of carrying a pathogenic *BRCA1/2* germline mutation by the statistical model BRCAPRO that considers: proband health state (un/affected); current age and age at diagnosis of the proband and all family members of four consecutive generations (only first/second degree relatives); typology of the existent tumors (unilateral/bilateral breast cancer, other cancers) [18]. Then, 69 patients with breast and thyroid cancer regardless of the sequence of appearance were recruited from Sant’Andrea Hospital and Regina Elena National Cancer Institute. Others 337 patients unselected cancer patients diagnosed at any age were recruited from Regina Elena National Cancer Institute.

### Cells and culture conditions

EBV-immortalized LCLs and freshly isolated PBMCs were cultured in RPMI-1640 GlutaMAX supplemented with 15 % heat-inactivated fetal bovine serum, 100 U/ml penicillin, and 100 μg/ml streptomycin (all from Invitrogen, CA, USA). PBMCs were isolated from donors’ heparinized blood samples by Lympholyte-H (Cedarlane, Burlington, USA) density gradient centrifugation. PBMCs were stimulated to proliferate by incubation with 5 μg/ml PHA (Sigma-Aldrich, St. Louis, MO, USA) and incubated at 37 °C in a 5 % CO_2_ atm for 60 h [14].

### p53-MCL test

Proliferating cells (i.e., LCLs or PHA-stimulated PBMCs) were set up for p53-MCL test as previously described [14]. Cells were examined under an Olympus BX53 microscope equipped with epifluorescence. Percentages of p53-MCL were measured by counting 100 cells in metaphase and analyzing two coverslips for each sample. The percentage of p53 mitotic localization is from 75 to 90 % for normal subjects, from 40 to 55 % for ATM carriers, and from 0 to 30 % for A-T patients.

### *BRCA1/2* gene sequencing

Genomic DNA was extracted from peripheral blood of all probands using commercial kit (Qiamp Blood Kit, Qiagen, Hilden, Germany). The entire coding sequence and each intron/exon boundary of *BRCA1* and *BRCA2* were screened by direct sequencing. All truncating and/or novel genetic variants were confirmed by sequencing different samples on both DNA strands. Sequencing was performed using the BigDye Terminator v3.1 Cycle Sequencing Kit and a 3130xl Genetic Analyzer (Applied Biosystems, CA, USA). Reference sequence for *BRCA1* was Genebank NM_007294.3, NG_005905.2 and reference sequence for *BRCA2* was Genebank, NM_000059.3, NG_012772.3. *BRCA1/2* genomic rearrangements were searched by the Multiple Ligation dependent Probe Amplification (MLPA) methodology. MLPA procedure was carried out according to the manufacturer’s instructions. Variations in peak height were evaluated comparing each sample with a normal control and by a cumulative comparison.

### *ATM* gene sequencing

Genomic DNA was extracted from PBMCs by Quick-gDNA MiniPrep (Zymo Research, CA, USA) according to the manufacturer’s instructions. Sixty-two ATM exons were amplified using AmpliTaq Gold (Applied Biosystems, CA, USA), as described [14], and subjected to direct sequencing at the Genechron Laboratory (Rome, Italy)**.** Reference sequence for *ATM* was Genebank U82828.1.

### *In silico* analysis

To predict possible impact of amino acid substitutions on the structure and function of human proteins, the PolyPhen-2 (Polymorphism Phenotyping v2) software, that uses straightforward physical and evolutionary comparative considerations [19], was employed.

Efficient splicing of many exons requires splicing-enhancers to promote splicing at unfavorable splice-sites or splicing-silencers to repress more favorable splice-sites nearby. As a consequence, single nucleotide changes in an exon or intron close to these splice-sites may be predicted to disrupt splicing. For *in silico* prediction of the effects of mutations on normal splicing, the interactive biosoftware Alamut v2.3 [20] was adopted.

### Statistic

We determined statistical significance of differences between two groups by 2-tailed Student’s t test. *P* values less than 0.01 were considered significant.

## Results

**Fig. 1:**
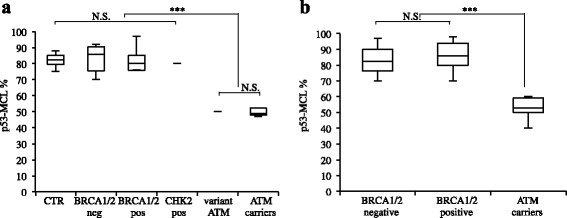
p53-MCL in LCLs and in PBMCs from familial breast and/or ovarian *BRCA1/2*-positive and –negative patients. (**a**) Comparison of p53-MCL percentages, among LCLs derived from wild type ATM donors (CTR, *n* = 11), BRCA1/2-negative (*n* = 7), BRCA1/2-positive (*n* = 5), CHK2-positive (*n* = 1), and ATM variants carriers (*n* = 2) and ATM carriers (*n* = 9) previously tested. Not significant differences among the groups including wild type ATM (CTR, BRCA1/2-neg, BRCA1/2-pos and CHK2-pos), but significant differences between these groups and ATM carriers groups (ATM variants and ATM carriers). (**b**) Comparison of p53-MCL percentages among PBMCs from familial breast and/or ovarian BRCA1/2-positive, BRCA1/2–negative and ATM carrier patients. Not significant differences between BRCA1/2-positive and -negative patients, but significant differences between wild type ATM group (BRCA1/2-positive and -negative) and ATM carriers. ***P < 0.0001; NS = Not Significant; 2-tailed Student’s t test

Beyond the high penetrance *BRCA1/2* genes, mutations of several cancer susceptibility genes, including *ATM*, *CHK2* and *PALB2*, have been shown to associate, with a moderate penetrance, with familial breast and/or ovarian cancers [21, 22]. Thus, we first verified p53-MCL test specificity compared to mutant BRCA1/2 and CHK2 proteins that are also involved in DNA damage response and centrosome amplification and localization [23–25]. LCLs from 15 familial breast and ovarian cancer (Table 1) including four BRCA1-positive, 2 BRCA2-positive, 1 CHK2-positive and 8 BRCA1/2/CHK2/PALB2-negative LCLs were analyzed by the p53-MCL test. As shown in Table 1 and Fig. 1a, the percentage of p53-MCL was compatible with that of wild-type cells in 13 out of 15 LCLs, while two cases showed a p53-MCL reduction typical of ATM carriers, one in a BRCA1-positive case and the other in a BRCA1/2/CHK2/PALB2-negative case. Direct *ATM* sequencing confirmed the presence of ATM variants (Table 1) in both cases demonstrating p53-MCL specificity for ATM variants also in comparison with mutations in the centrosome related factors BRCA1/2 and CHK2.

**Table 1:** Genetic status and p53-MCL rate of LCLs from familial breast and ovarian cancer

	*BRCA1*	*BRCA2*	*CHK2*	*PALB2*	*ATM*	p53-MCL%
BR36	wt	mut	nt	nt	nt	80
BR13	wt	mut	nt	nt	nt	76
BR409-3	mut	wt	nt	nt	nt	97
BR324-1	mut	wt	nt	nt	nt	85
BR404-1	mut	wt	wt	wt	wt	76
BR317	mut	wt	nt	nt	mut	50
BR377	wt	wt	wt	wt	mut	50
BR60-1	wt	wt	wt	wt	wt	71
BR107-1	wt	wt	wt	wt	wt	90
BR362-1	wt	wt	wt	wt	wt	92
BR494	wt	wt	wt	wt	wt	86
BR38	wt	wt	wt	wt	wt	91
BR48	wt	wt	wt	wt	wt	70
BR278-1	wt	wt	wt	wt	wt	80
BR501	wt	wt	mut	wt	wt	80

*wt* wild type, *mut* mutated, *nt* not tested

Next, we analyzed 90 cases of familial breast and/or ovarian cancer (Table 2). All 90 patients were screened for mutations in the *BRCA1* and *BRCA2* genes and 20 were found to carry pathogenic variants with an overall mutation rate of 22.2 % (Table 2). In particular BRCA1 pathogenic mutation recurred in about 26 % (8/30) of the Hereditary Breast and Ovarian Cancer (HBOC) families and in about 7 % (4/60) of the Hereditary Breast Cancer (HBC) families, while BRCA2 pathogenic mutation occurred in about 13 % (4/30) and 7 % (4/60) of the HBOC and HBC families, respectively. Of the 20 mutation-positive probands, 11 had breast cancer alone (5 *BRCA1* and 6 *BRCA2*), three had ovarian cancer alone (3 *BRCA1*), three had bilateral breast cancer (2 *BRCA1* and 1 *BRCA2*), two had both breast and ovarian cancer (1 *BRCA1* and 1 *BRCA2*) and one had both bilateral breast and ovarian cancer (*BRCA1*). When fresh PBMCs from *BRCA1/2*-positive and -negative patients were analyzed by the p53-MCL test, five out of 90 cases showed aberrant p53-MCL (Fig. 1b). Interestingly p53-MCL positive cases accounted for 6.6 and 5 % of the HBOC and HBC families, respectively (Table 2). Of note, this rate is similar to that of BRCA2 mutations [26].

**Table 2:** Characteristics of familial breast and/or ovarian cancer patients

Variable	Study population *n* = 90	BRCA1/2 carriers *n* = 20/90 (22.2 %)	ATM carriers *n* = 5/90(5.5 %)
Age-years			
Median	52.9	53.7	44.4
Range	30–82	40–75	32–51
Familial aggregation			
HBOC	30 (33.3 %)	BRCA1: 8/30 (26 %)	2/30 (6.6 %)
		BRCA2: 4/30 (13 %)	
HBC	60 (66.7 %)	BRCA1: 4/60 (7 %)	3/60 (5 %)
		BRCA2: 4/60 (7 %)	
BRCAPRO	27.3 %	50.1 %	52.2 %

*HBOC* hereditary breast and ovarian cancer, *HBC* hereditary breast cancer

**Fig. 2:**
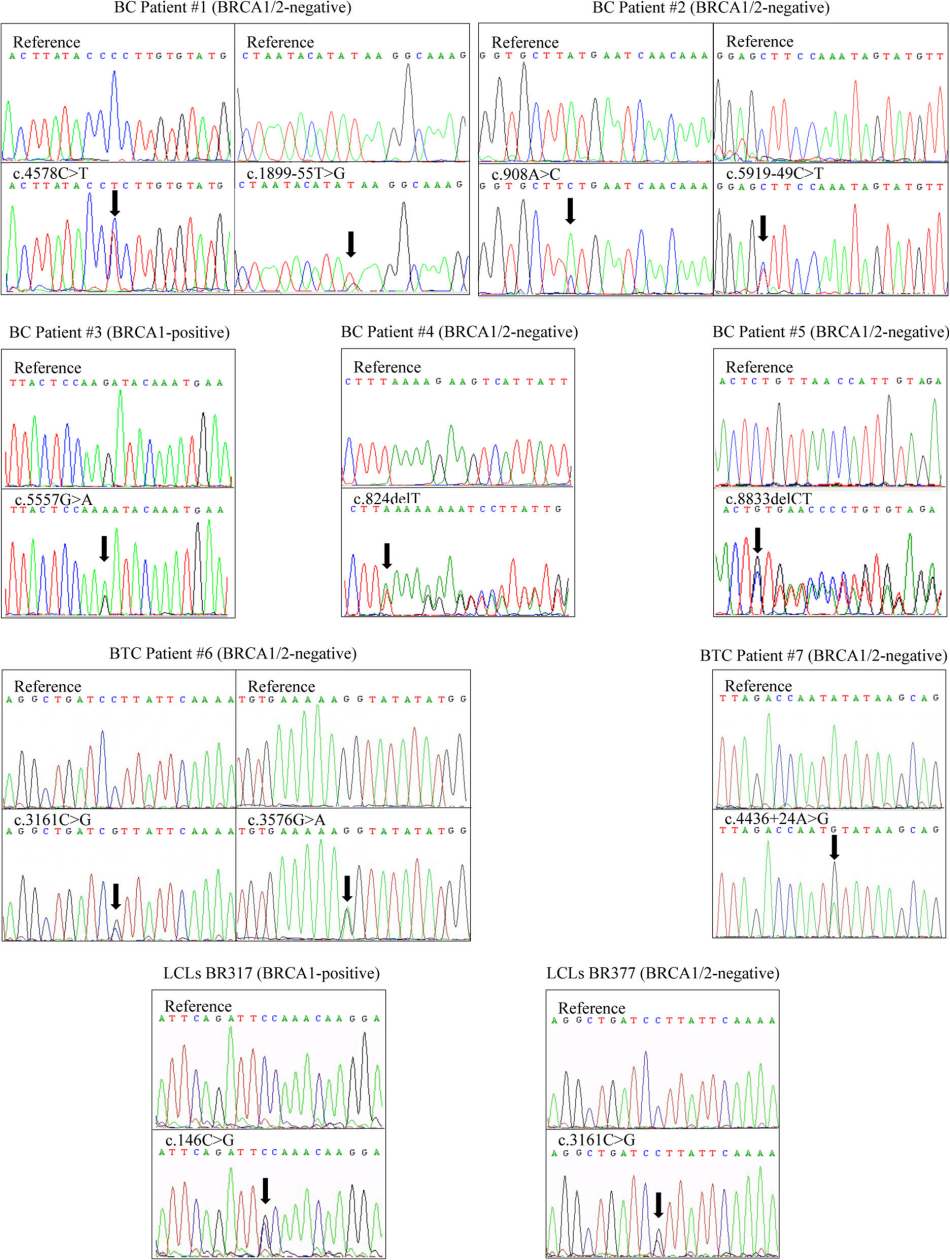
ATM variants in ATM carriers. Electropherograms showing ATM variants identified in PBMCs derived from five familial breast cancer (BC) patients (BC patient#1/#5), two breast-thyorid cancer (BTC) patients (BTC patient#6/#7) and two LCLs (BR317; BR377). All sequences are compared with wild-type reference sequence. *Arrows* indicates the position of the substitution and/or deletion

The five individuals with ATM variants identified by the p53-MCL test were all breast cancer patients (5/65, 7.7 %). Four of them were *BRCA1/2* negative patients and one was a *BRCA1* positive patient (Fig. 2). No ATM carriers were detected by p53-MCL in patients with ovarian cancer, bilateral breast cancer, or multiple cancers (breast cancer and at least one other non-breast cancer) (*n* = 27) (Table 3).

**Table 3:** Histopathological characteristics of all cancer patients

Tumor type	Cases *n* = 496	BRCA1/2 carriers *n* = 20	ATM carriers *n* = 7
Familial Cancer cases	90	20	5
Breast	65 (70.6 %)	11/65 (16.9 %)	5/65 (7.7 %)
Ovarian	8 (8.7 %)	3/8 (37.5 %)	0/8
Bilateral Breast	10 (10.9 %)	3/10 (30 %)	0/10
Breast-Ovarian	4 (4.3 %)	2/4 (50 %)	0/4
Colon-Breast	1 (1.1 %)	0/1	0/1
Uterin-Breast	1 (1.1 %)	0/1	0/1
Anal-Bilateral Breast	1 (1.1 %)	0/1	0/1
Bilateral Breast-Ovarian	1 (1.1 %)	1/1 (100 %)	0/1
Ipsilateral Breast	1 (1.1 %)	0/1	0/1
Sporadic cancer cases	406	-	2/406
Ovarian	49	-	0/49
Lung	150	-	0/150
Colon	80	-	0/80
Post-menopausal Breast	58	-	0/58
Breast/Thyroid	69	-	2/69 (2.9 %)

Besides early onset-breast cancer, ATM carriers have been reported to be more susceptible to other types of cancer, such as digestive tract, lung, and thyroid cancers [2, 8]. Thus, we performed the p53-MCL test on fresh PBMCs from 403 patients with sporadic cancers, including ovarian (*n* = 49), lung (*n* = 150), colon (*n* = 80), post-menopausal breast (*n* = 58), and both breast/thyroid (*n* = 69) cancer. Two individuals with ATM variants were identified among breast/thyroid cancer cases (2/69, 2.9) (Table 4). No ATM carriers were found in sporadic ovarian cancer (0/49), non-small cell lung cancer (0/150), colorectal cancer (0/80), and post-menopausal breast cancer (0/58).

**Table 4:** Clinical and pathological characteristics of breast and thyroid cancer patients

Variable	Study population (*n* = 69)	p53-MCL positive (*n* = 2)
Age-years		
Average	60.2	58
Range	30–77	58–63
Age onset Breast tumor appearance		
Average	49.1	48
Range	25–70	48–51
Breast tumor Diagnosis		
Ductal	59 (85.5 %)	1 (50 %)
Lobular	3 (4.3 %)	0
Unknown	7 (10.2 %)	1 (50 %)
ER status		
Negative	17 (24.6 %)	0
Positive	34 (49.3 %)	2 (100 %)
Unknown	18 (26.1 %)	0
PgR status		
Negative	17 (24.6 %)	0
Positive	34 (49.3 %)	2 (100 %)
Unknown	18 (26.1 %)	0

*ER* estrogen receptor, *PgR* progesterone receptor

**Fig. 3:**
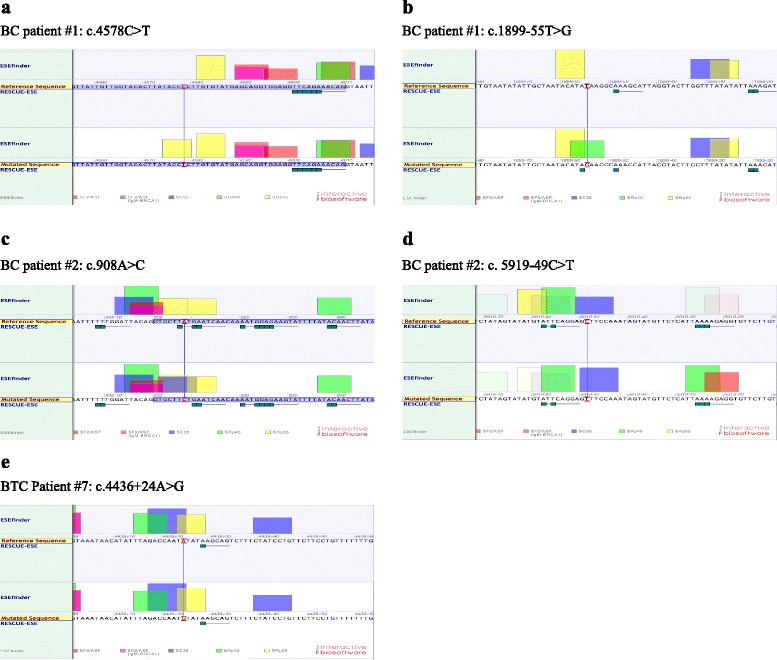
In silico analysis using Alamut software. ATM variants identified in PBMCs derived from two familial breast cancer patients (BC patient#1 and #2) and one breast-thyorid cancer patient (BTC patient#7) lead to an increased affinity and/or appearance of a de novo sites for SR proteins

As shown in Fig. 2 and Additional file 1: Table S1, 11 different ATM variants were detected among the 9 p53-MCL positive cases (*i.e*., 2 LCLs and 7 PBMCs). Specifically, in breast cancer cases, patient #1 presents c.4578C > T and c.1899-55 T > G variants. The c.4578C > T was previously described [27] and corresponds to a synonymous substitution (P1526P), which is predicted to create a new exonic site with increased affinity for the SRp55 splicing factor by an *in silico* analysis (Fig. 3a). The c.1899-55 T > G is a previously described intronic variant [28] predicted to increase the affinity for the SRp40 splicing factor (Fig. 3b). Patient #2 presents two variants not previously described in the literature nor in the ATM variation database (c.908A > C and c.5919-49C > T). Our *in silico* analyses predict a higher affinity for the splicing factor SC35, for both variants (Fig. 3c, d). The c.5557G > A (D1853N) variant presents in patient #3 is predicted to be “possibly damaging” by the PolyPhen-2 tool and has been intensively studied with respect to its possible association with breast cancer susceptibility [29]. Patient #4 and patient #5 present two deleterious variants, c.824delT and c.8833delCT, that induce early protein truncation at the level of exon 9 and 63, respectively, which have already been described in ATM families [30, 31]. In the breast and thyroid cancer cases, patient #6 presents two already described variants, c.3161C > G and c.3576G > A. The c.3161C > G (P1054R) is a substitution predicted to be “*possibly damaging”* by PolyPhen-2 and implicated in breast and prostate cancer risk [32]. The c.3576G > A variant leads to exon 26 skipping and causes A-T syndrome in homozygosis [30]. Patient #7 presents the intronic variant c.4436 + 24A > G that *in silico* analyses predict a mild higher affinity for the splicing factors SC35 and SRp55 (Fig. 3e). LCLs BR317 presents c.146C > G (S49C) variant, a substitution predicted to be possibly damaging by PolyPhen-2 and reported as breast cancer susceptibility variant [16]. Finally, LCLs BR377 presents the same c.3161C > G (P1054R) variant found in patient #6.

## Discussion

Genetic susceptibility plays an important role in several common chronic diseases including many types of cancer. Genetic testing for large-scale, general population screening can be very expensive and non-cost effective for National Health Services. Concerning the *ATM* gene, the complex genomic organization, the large number of polymorphisms, the absence of mutation hot-spots, and the frequent occurrence of variants of yet uncharacterized but predicted deleterious functions, make direct gene sequencing not yet a cost-effective approach. This is particularly evident for large-scale surveys of mutant/variant ATM carriers for which gene sequencing is not sufficient to classify the rare hits. All these factors hinder both genetic counseling and clinical guidelines for risk management of ATM carriers and their families. The use of the recently developed p53-MCL functional test to detect ATM carriers might overcome at least some of these limitations. Here, we established the p53-MCL specificity for *ATM* in respect to the high-risk, *BRCA1/2* breast/ovarian cancer susceptibility genes and confirmed p53-MCL as reliable test to detect variant ATM carriers in cancer patients.

BRCA1/2 and ATM proteins share functional activities both in DNA damage response pathways and in centrosome regulation [33]. Thus, it was mandatory to establish whether a functional test, such as the p53-MCL test, would discriminate between *ATM* and *BRCA1/2* variant carriers. Analysis of *BRCA1/2*-positive LCLs (*n* = 6) and PBMCs (*n* = 20), and of 1 *CHK2*-positive LCL established the specificity of p53-MCL test on *ATM* in respect to *BRCA1/2* and CHK2 mutations (Table 1 and Fig. 1).

A total of seven individuals with ten ATM variants were identified by p53-MCL test in PBMCs: five breast cancer patients in HBC and HBOC families and two breast/thyroid cancer patients. The finding that all cases were among pre-menopausal breast cancer cases but not among patients with post-menopausal breast cancer or ovarian cancer is consistent with previous studies showing prevalence of germline ATM mutations detected by sequencing in patients with familial early-onset breast cancer [34] or by p53-MCL in sporadic breast cancer [14]. Interestingly, the a priori probability of carrying a pathogenic *BRCA1/2* germline mutation measured by the BRCAPRO score showed a mean for the entire cohort of 27.3 while the mean for the five ATM carriers was 52.2 % (Table 2). This difference is statistically significant (*P =*0.01) and supports the presence of a strong genetic component in the ATM carriers with early-onset breast cancer, also considered that the BRCAPRO score for the BRCA positive cases is very similar to ATM positive ones (50.1 % vs. 52.2 %).

Evaluation of the histopathologic features of the breast cancers developed in the seven identified ATM carriers showed the presence of estrogen and progesterone receptors and the absence of HER2 receptor in four out of five cases that have been tested (Tables 4 and 5), confirming our previous results [14]. Thus, the presence of germline ATM variants recognized by p53-MCL appears to identify a subset of tumors with a more favorable biomarker asset, despite their earlier onset. In addition, a possible association between breast and thyroid cancer was highlighted by the identification of two ATM carriers among the sporadic cancer patients with breast/thyroid cancer (2/69, 2.9 %), encouraging for further studies.

**Table 5:** Clinical and pathological characteristics of breast cancer patients of HBOC and HBC

Variable	Study population (*n* = 65)	p53-MCL positive (*n* = 5)
Age-years		
Average	50.4	44.4
Range	30–82	32–51
Age onset tumor appearance		
Average	42.5	38.6
Range	24–67	28–43
Diagnosis		
Ductal	47 (72.3 %)	3 (60 %)
Lobular	8 (12.3 %)	0
Unknown	8 (12.3 %)	2 (40 %)
Other	2 (3.1 %)	0
ER status		
Negative	15 (23.1 %)	0
Positive	36 (55.4 %)	5 (100 %)
Unknown	14 (21.5 %)	0
PgR status		
Negative	19 (29.2 %)	0
Positive	32 (49.2 %)	5 (100 %)
Unknown	14 (21.5 %)	0
HER2 status		
Negative	29 (44.6 %)	4 (80 %)
Positive	16 (24.6 %)	1 (20 %)
Unknown	20 (30.8 %)	0
BRCAPRO	32.3	52.2

*ER* estrogen receptor, *PgR* progesterone receptor, *HER2* human epidermal growth factor receptor 2

Familial breast and ovarian cancers are linked to highly penetrant mutations in the *BRCA1*/*2* susceptibility genes that overall account for 20–25 % of hereditary breast cancers and 15 % of ovarian cancers [26]. Gene panel next generation sequencing approaches identified moderate-penetrant mutations in the *ATM* gene in 2.9 % [11] and 2.3 % [34] of *BRCA1/2*-negative cases. By the p53-MCL test, in this study we found 5.5 % (5/90) of ATM mutation carriers, which adds up to the 27.7 % (20/90) carrying BRCA1/2 mutations. In particular, three ATM mutations occurred among the 60 HBC cases, with a mutation rate (5 %) very close to that observed for BRCA2 (7 %) in the same subset. These observations suggest that the fast and non-expensive p53-MCL test should precede or be performed in parallel with BRCA1/2 sequencing.

Most of the ATM mutations occurring in A-T patients are frameshift or nonsense mutations leading to protein truncation or splice junction variants [6, 7] The role of A-T-causing mutations in cancer susceptibility (in particular breast cancer) is still debated and some studies have shown that a subset of rare, evolutionarily unlikely missense substitutions are important [15, 35, 36]. Here, the p53-MCL test identified 11 ATM variants in 9 breast and breast/thyroid patients. Three variants (c.824delT; c.8833delCT; c.3576G > A) identified in 3 different patients are known to cause A-T in homozigosity [30, 31]. Other 3 variants (c.5557G > A; c.146C > G; c.3161C > G) identified in 3 different patients have been shown to be associated with an increased cancer risk [16, 29, 32]. The last 3 patients present 5 different variants (c.4578C > T; c.1899-55 T > G; c.908A > C; c.5919-49C > T; c.4436 + 24A > G) that our *in silico* analysis predicted to increase the affinity for splicing factor and modify alternative splicing activities. Whether these predicted modifications alter ATM function/s and cancer predisposition remain to be evaluated. Since loss of p53 centrosomal localization was the functional readout through which we diagnosed the ATM carriers, we can conclude that these variants are at least able to impair the mitotic localization of p53 at the centrosomes. It will be relevant to study whether all ATM variants induce this p53 defect or whether only functionally relevant variants are able to impair p53-MCL. Whether this impaired p53 localization has a role in tumorigenesis is presently unknown. Studying the mechanistic basis of p53 centrosomal localization will give insights on the contribution that different ATM variants with uncertain significance might have in cancer predisposition. Application of the p53-MCL test to LCLs or PBMCs with a broad spectrum of ATM variants will help to define these issues.

## Conclusions

Our results show that p53-MCL test may offer the opportunity for screening of the general population and to identify the differences among deleterious, neutral and beneficial variants helping, in the future, to define the guidelines for ATM carriers not only in the A-T families.

## Additional file

Additional file 1: Table S1. Characteristics of ATM variants. (DOCX 75 kb)

## Abbreviations

A-T: Ataxia telangiectasia
ATM: Ataxia telangiectasia mutated
ATR: Ataxia telangiectasia and Rad3 related
BRCA: Breast cancer susceptibility gene
CHK2: Checkpoint kinase 2
EBV: Epstein barr virus
FANC-A: Fanconi anemia complementation group A
HBC: Hereditary breast cancer
HBOC: Hereditary breast and ovarian cancer
LCLs: Lymphoblastoid cell lines
MRE11: Meiotic recombination 11
NBS1: Nijmegen breakage syndrome 1
p53-MCL: p53-Mitotic centrosomal localization
PALB2: Partner and localizer of BRCA2
PBMCs: Peripheral blood mononuclear cells
PHA: Phytohemagglutinin
SMC1A: Structural maintenance of chromosomes 1A
WRN: Werner syndrome
