# Erratum to: Detection of *ATM* germline variants by the p53 mitotic centrosomal localization test in *BRCA1/2*-negative patients with early-onset breast cancer

**DOI:** 10.1186/s13046-016-0459-z

**Published:** 2016-11-28

**Authors:** Andrea Prodosmo, Amelia Buffone, Manlio Mattioni, Agnese Barnabei, Agnese Persichetti, Aurora De Leo, Marialuisa Appetecchia, Arianna Nicolussi, Anna Coppa, Salvatore Sciacchitano, Carolina Giordano, Paola Pinnarò, Giuseppe Sanguineti, Lidia Strigari, Gabriele Alessandrini, Francesco Facciolo, Maurizio Cosimelli, Gian Luca Grazi, Giacomo Corrado, Enrico Vizza, Giuseppe Giannini, Silvia Soddu

**Affiliations:** 1 Unit of Cellular Networks and Molecular Therapeutic Targets, Department of Research, Advanced Diagnostic, and Technological Innovation, Regina Elena National Cancer Institute – IRCCS, Via Elio Chianesi 53, 00144 Rome, Italy; 2 Istituto Pasteur-Fondazione Cenci Bolognetti, Department of Molecular Medicine, University La Sapienza, Rome, Italy; 3 Endocrinology Unit, Department of Clinical and Experimental Oncology, Regina Elena National Cancer Institute – IRCCS, Rome, Italy; 4 Department of Molecular Medicine, University La Sapienza, Rome, Italy; 5 Department of Clinical and Molecular Medicine, University La Sapienza, Laboratorio di Ricerca Biomedica, Fondazione Università Niccolò Cusano per la Ricerca Medico Scientifica, Rome, Italy; 6 Radiotherapy Unit, Department of Research, Advanced Diagnostic, and Technological Innovation, Regina Elena National Cancer Institute - IRCCS, Rome, Italy; 7 Medical Physics Unit, Department of Research, Advanced Diagnostic, and Technological Innovation, Regina Elena National Cancer Institute – IRCCS, Rome, Italy; 8 Toracic Surgery Unit, Department of Clinical and Experimental Oncology, Regina Elena National Cancer Institute – IRCCS, Rome, Italy; 9 Hepato-pancreato-biliary Surgery Unit, Department of Clinical and Experimental Oncology, Regina Elena National Cancer Institute – IRCCS, Rome, Italy; 10 Gynecological Oncology Unit, Department of Clinical and Experimental Oncology, Regina Elena National Cancer Institute – IRCCS, Rome, Italy; 11 Department of Experimental Medicine, Sapienza University of Rome, Policlinico Umberto I, Viale Regina Elena, 32400161 Rome, Italy

## Erratum

In original publication of this article [1], the data for “Pat#7” in Additional file 1 was listed as “c.4436+24G>A” in the “Nucleotide change” column. Instead, the number should have been c.4436+24A>G.

## Additional file

Additional file 1: Characteristics of ATM variants. (DOCX 16 kb)
