# Supplementary material for: Detection of ATM germline variants by the p53 mitotic centrosomal localization test in BRCA1/2-negative patients with early-onset breast cancer
Source: J Exp Clin Cancer Res. 2016 Sep 6;35(1):135. doi: 10.1186/s13046-016-0410-3 (PMC5012020; doi:10.1186/s13046-016-0410-3)
Supplement: Additional file 1: Table S1. — Characteristics of ATM variants. (DOCX 75 kb) [file 13046_2016_410_MOESM1_ESM.docx]

Table S1

Characteristics of ATM variants

| **Case** | **Nucleotide change** | **Amino acid change** | **Exon/IVS** |  | **Variant** | **Polyphen-2** | **Alamut** | **BRCA1/2** | **Ref** | **Tumor** |
| --- | --- | --- | --- | --- | --- | --- | --- | --- | --- | --- |
|  |  |  |  |  |  |  |  |  |  |  |
| **Pat#1** | **c.4578C>T**  **c.1899-55T>G** | p.P1526P  - | Ex32  IVS14 |  | S  U | N/T  N/T | SRp55  SRp40 | negative | 26  27 | B |
| **Pat#2**  **Pat#3**  **Pat#4**  **Pat#5**  **Pat#6**  **Pat#7**  **LCL#317**  **LCL#377** | **c.908A>C**  **c.5919-49C>T**  **c.5557G>A**  **c.824delT**  **c.8833delCT**  **c.3161G>C**  **c.3576G>A**  **c.4436+24G>A**  **c.146C>G**  **c.3161G>C** | p.Y303S  -  p.D1853N  p.L275*  p.L2945fs  p.P1054R  p.S135_K1192del58  -  p.S49C  p.P1054R | Ex10  IVS41  Ex39  Ex9  Ex63  Ex24  Ex26  IVS32  Ex5  Ex24 |  | M  U  M  T  T  M  D  U  M  M | Benign  N/T  Prob dam  N/T  N/T  Prob dam  N/T  N/T  Poss dam  Prob dam | SC55  SC35  N/T  N/T  N/T  N/T  N/T  #  N/T  N/T | negative  BRCA1  negative  negative  negative  negative  BRCA1  negative | -  -  28  29  30  31  29  -  16  31 | B  B  B  B  B/Th  B/Th  B/Th  B  B |

Pat, patient. Ex, exon; IVS, intron. S, synonymous variant; U, unclassified variant; M, missense variant; T, truncating variant; D, in-frame deletion variant. N/T, not tested; Prob dam, probably damaging; Poss dam, possibly damaging; SRp55, SRp40, SC35, SC55, splicing factors; Ref, reference; B, breast; Th, thyroid
